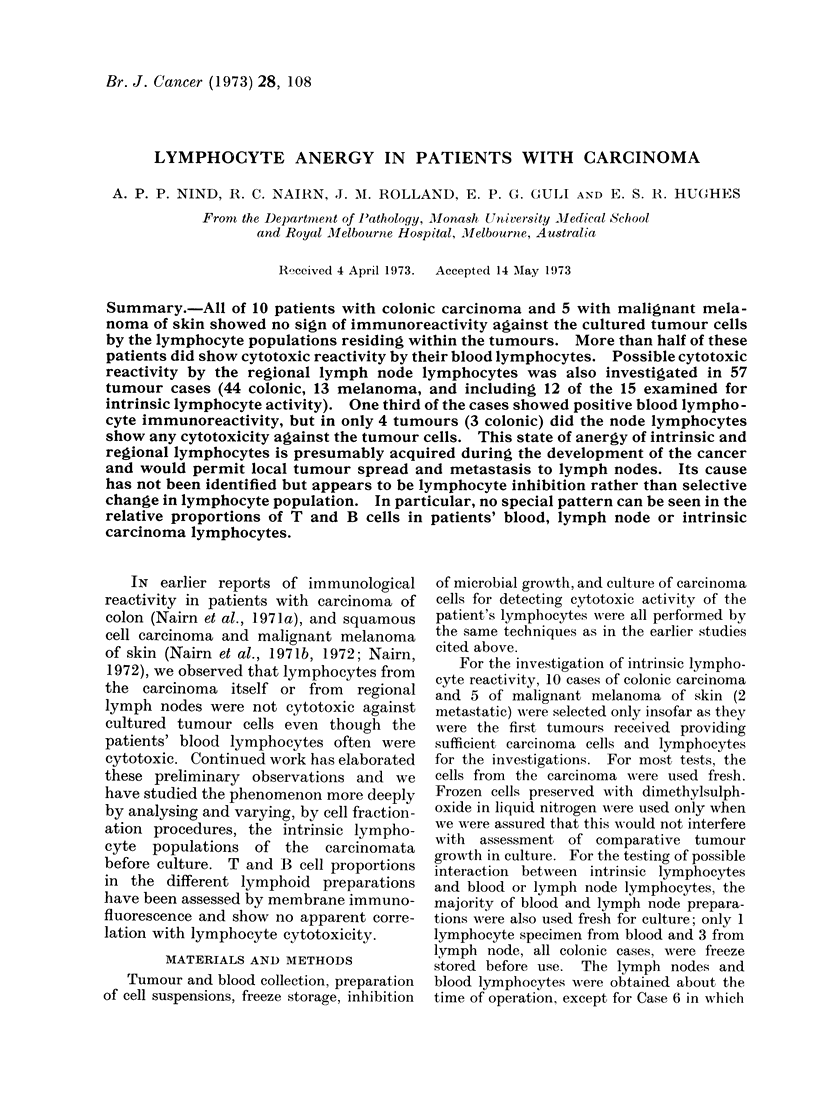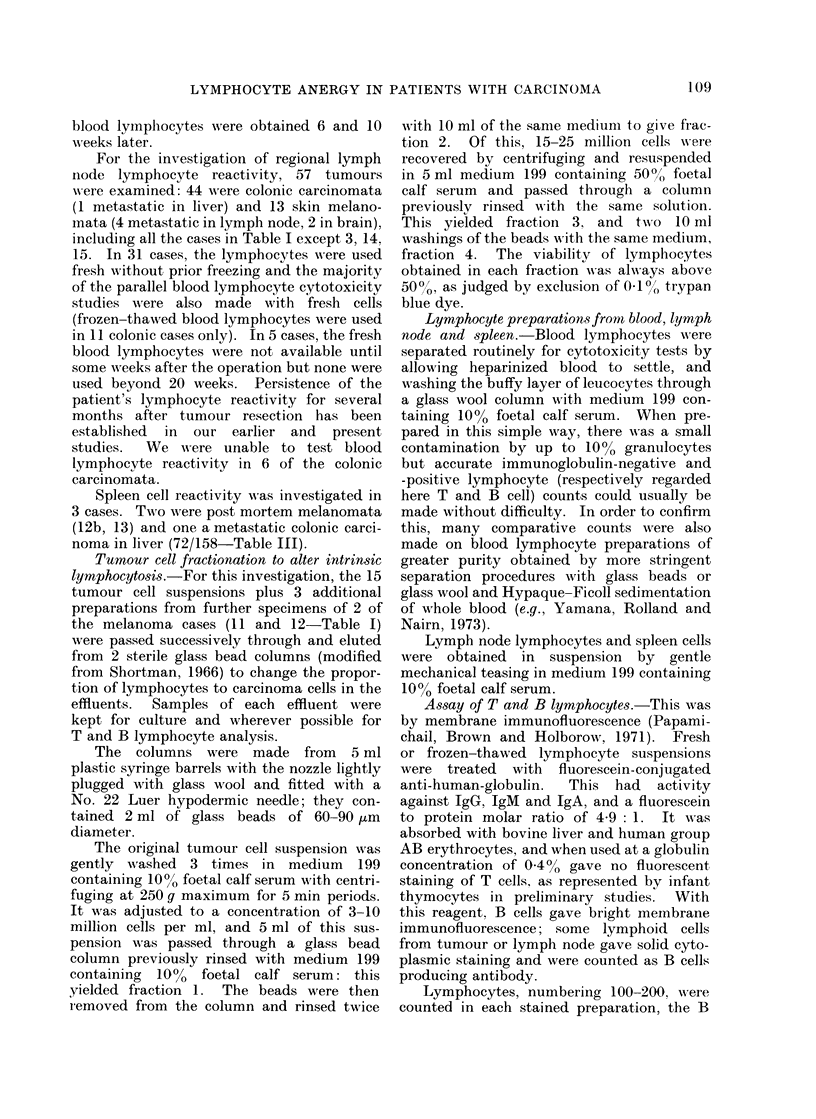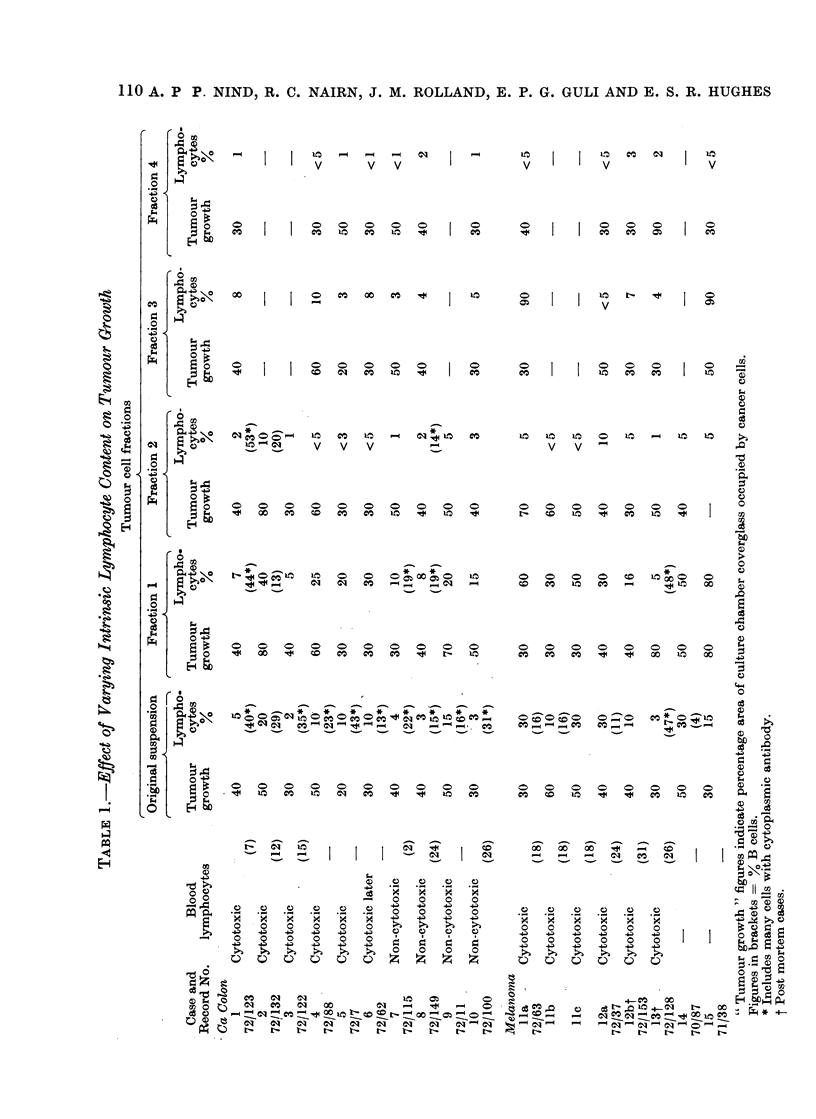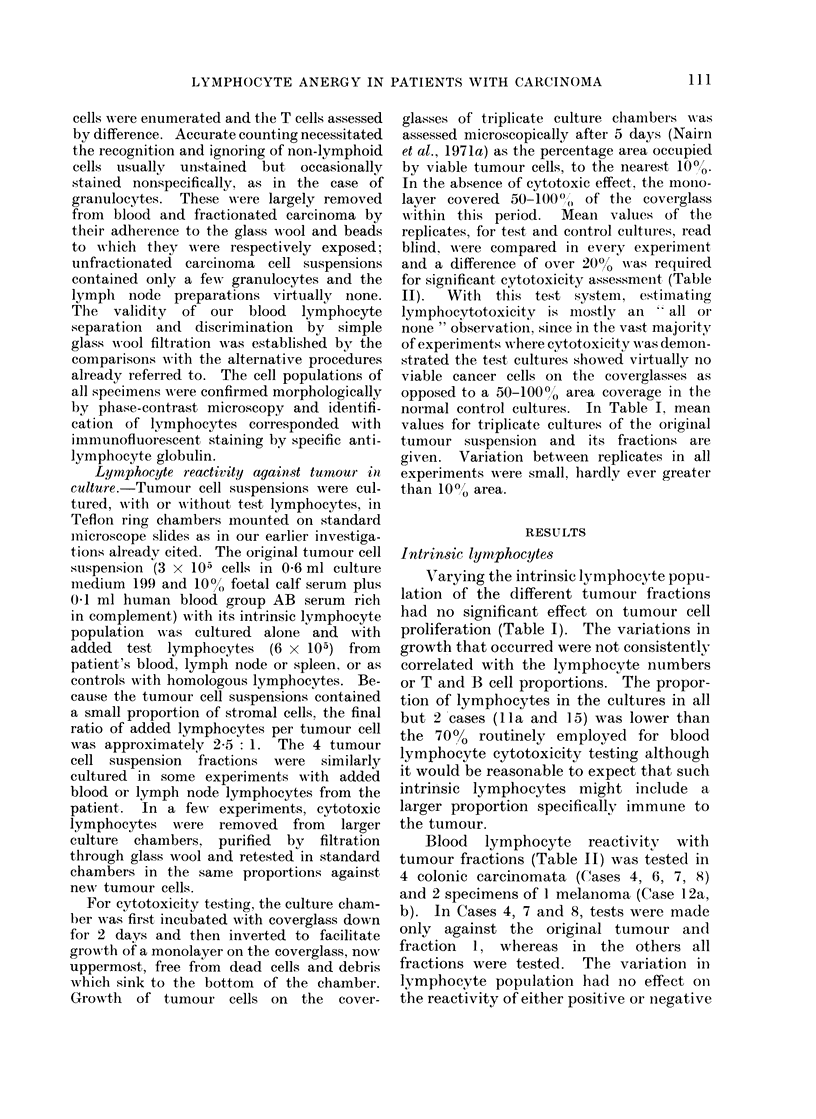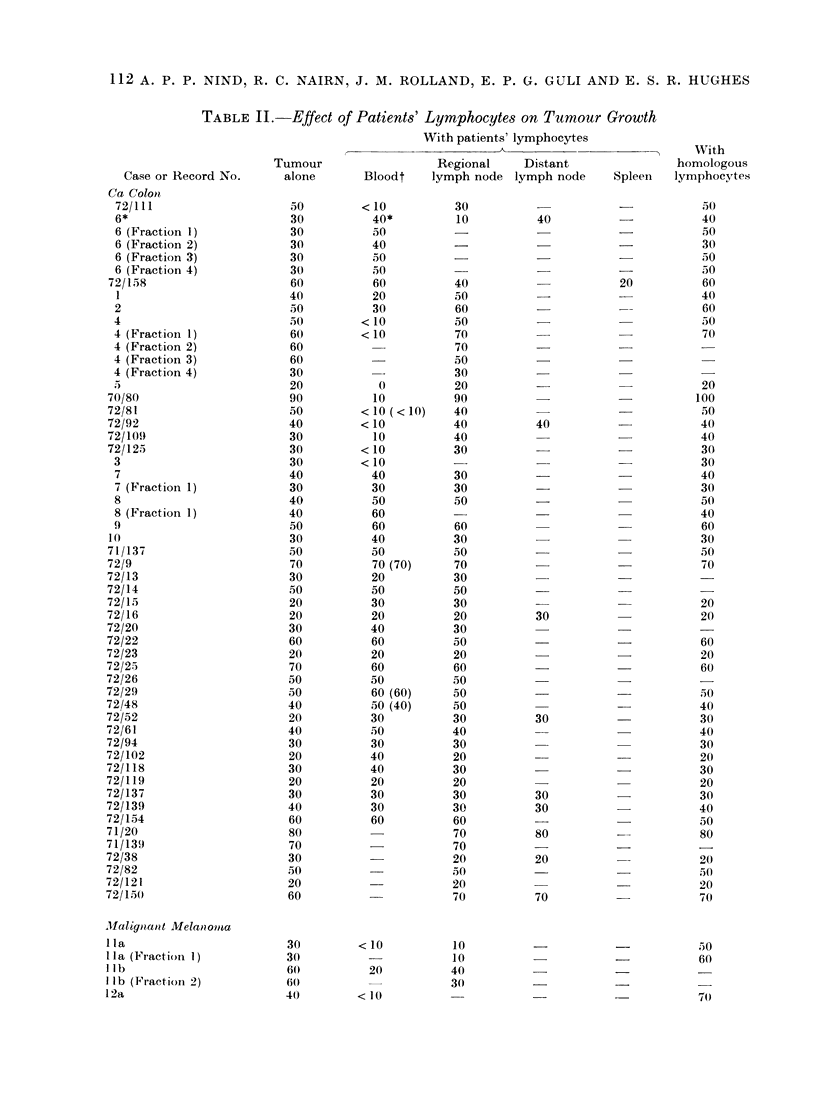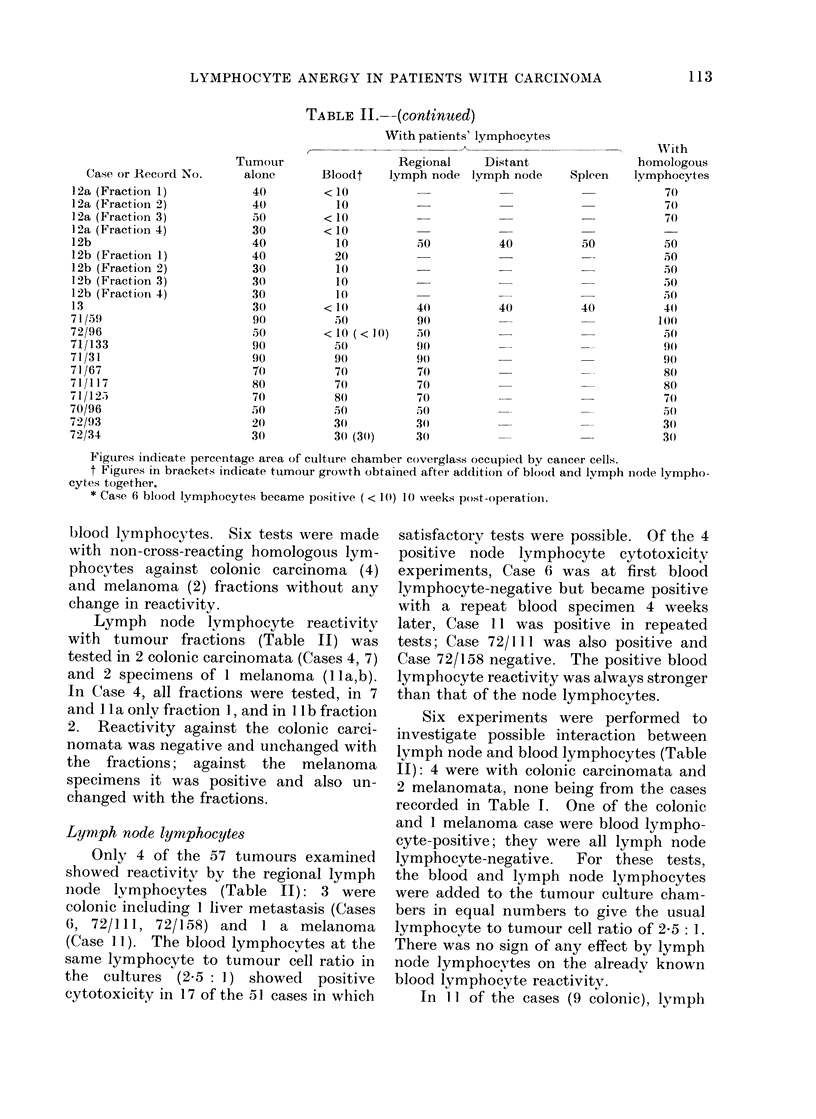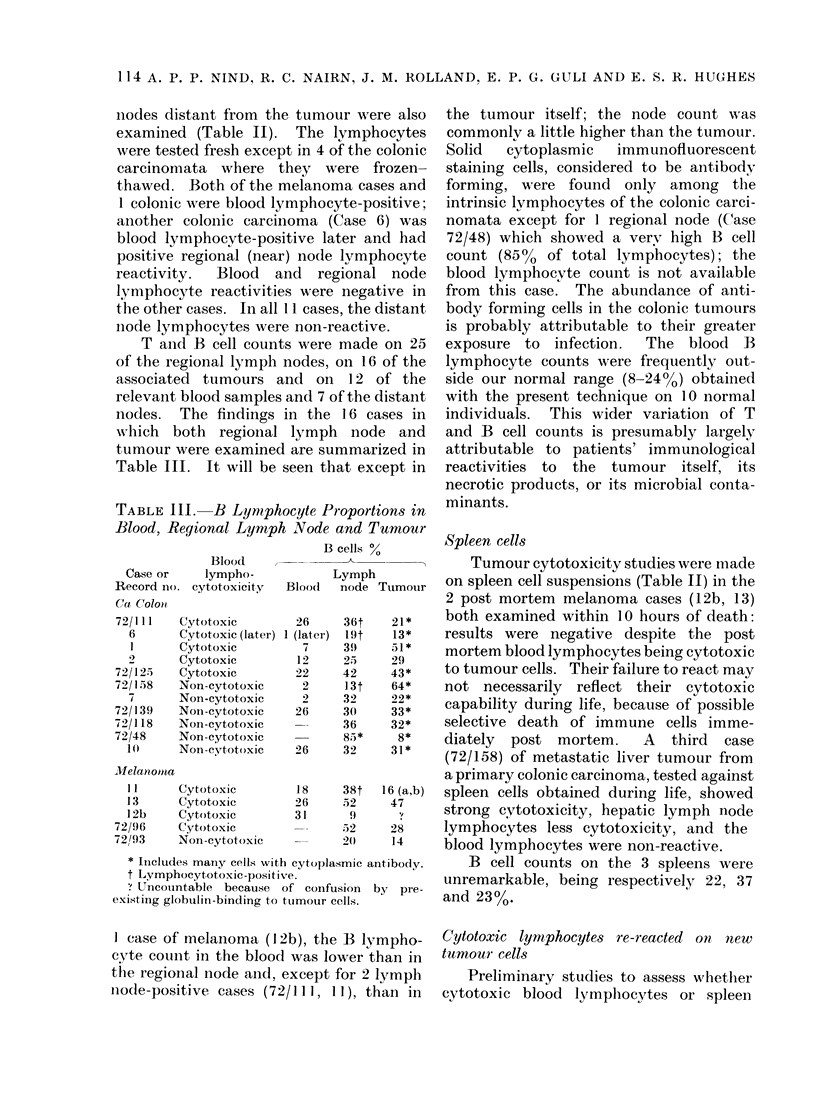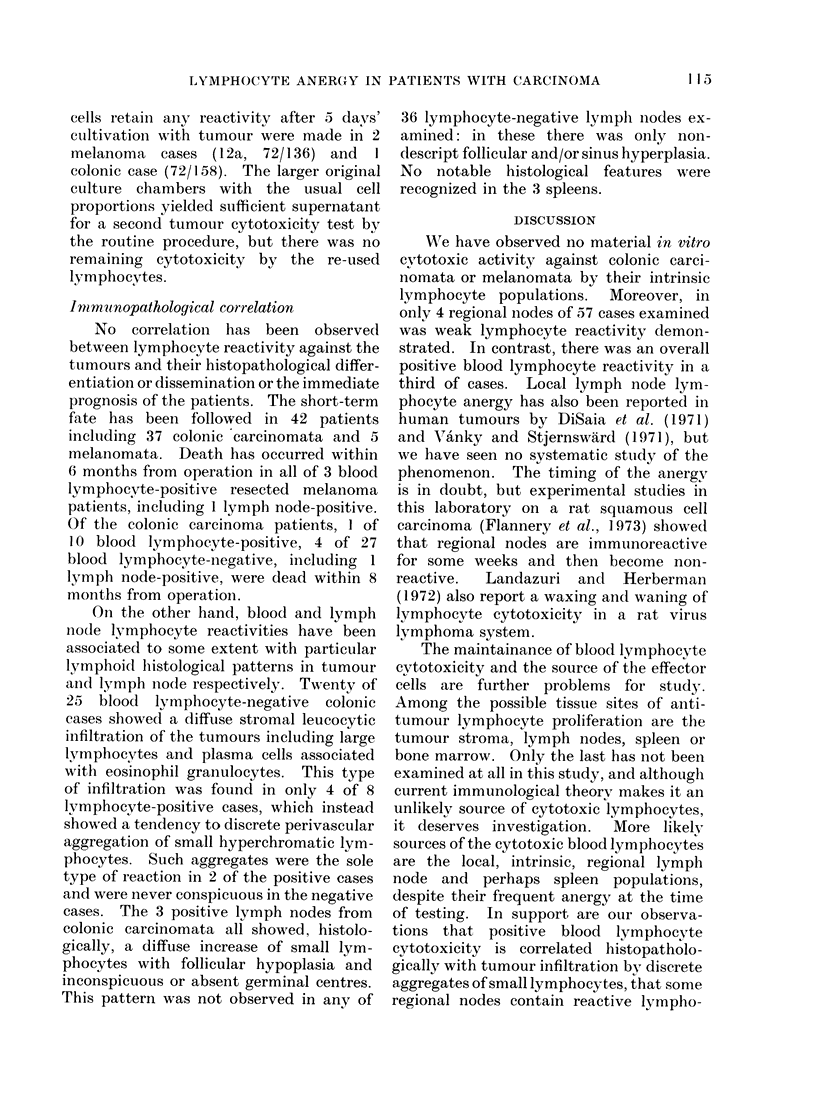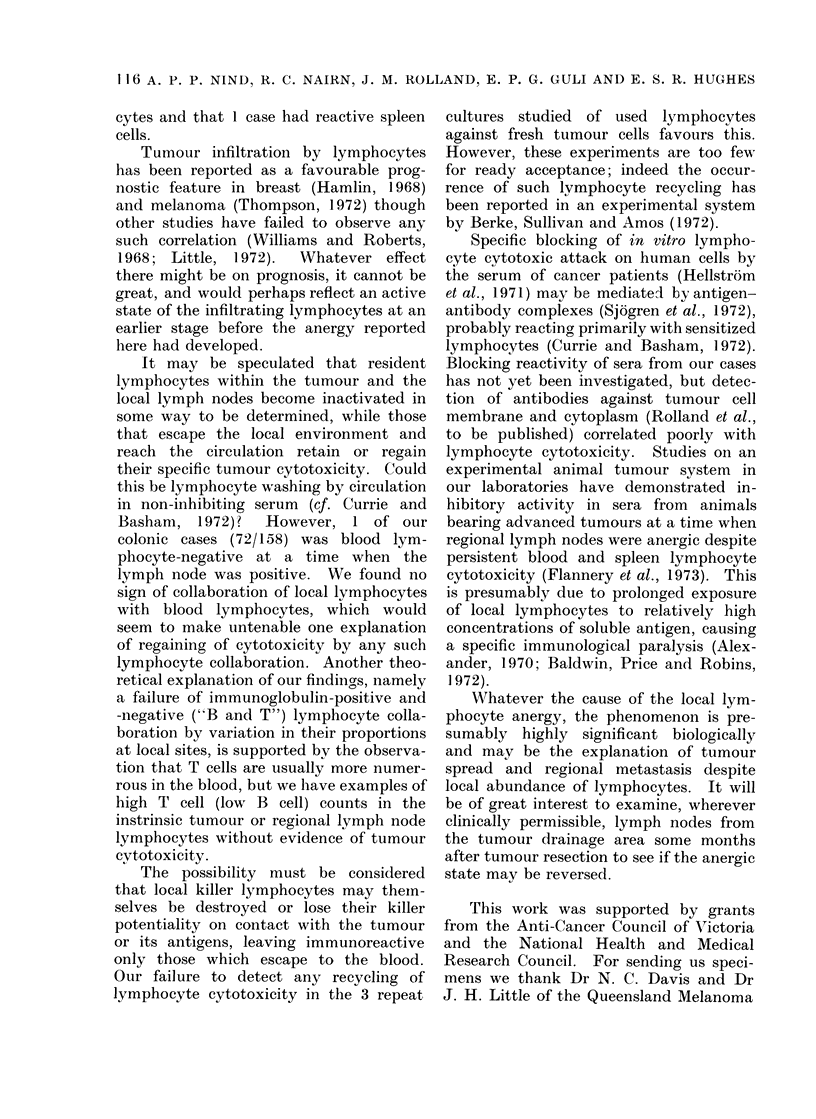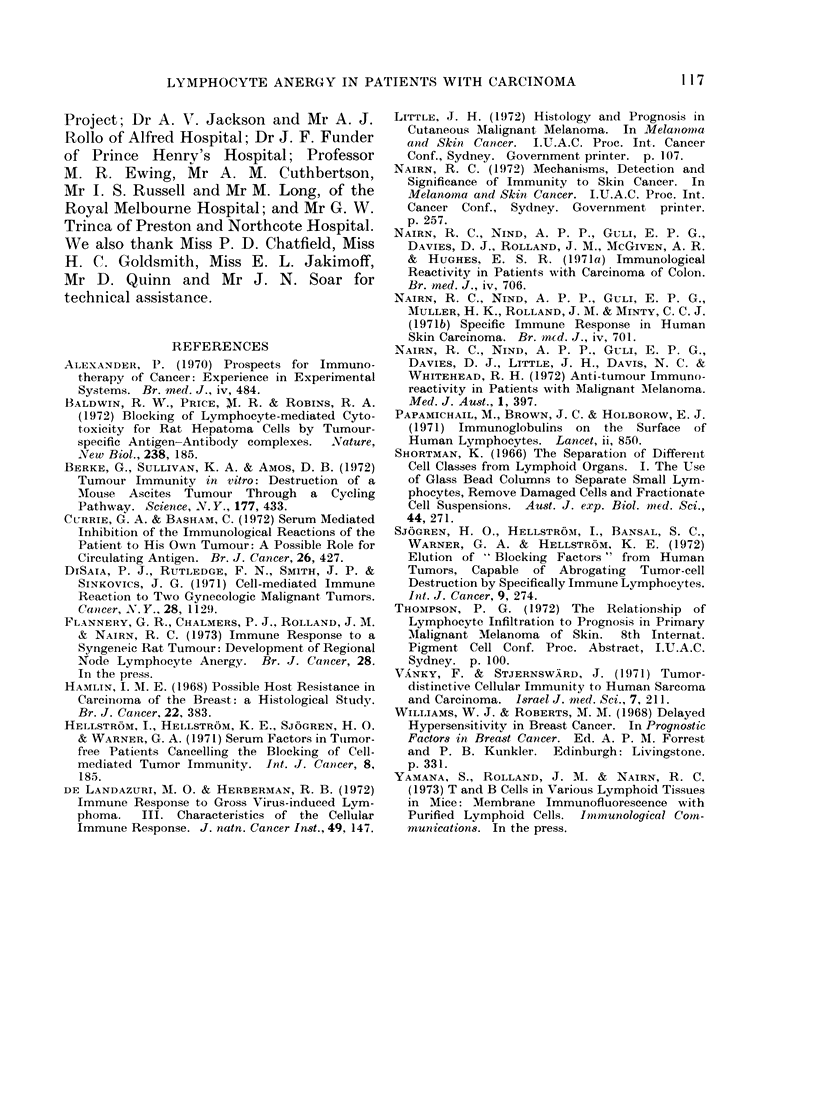# Lymphocyte Anergy in Patients with Carcinoma

**DOI:** 10.1038/bjc.1973.128

**Published:** 1973-08

**Authors:** A. P. P. Nind, R. C. Nairn, J. M. Rolland, E. P. G. Guli, E. S. R. Hughes

## Abstract

All of 10 patients with colonic carcinoma and 5 with malignant melanoma of skin showed no sign of immunoreactivity against the cultured tumour cells by the lymphocyte populations residing within the tumours. More than half of these patients did show cytotoxic reactivity by their blood lymphocytes. Possible cytotoxic reactivity by the regional lymph node lymphocytes was also investigated in 57 tumour cases (44 colonic, 13 melanoma, and including 12 of the 15 examined for intrinsic lymphocyte activity). One third of the cases showed positive blood lymphocyte immunoreactivity, but in only 4 tumours (3 colonic) did the node lymphocytes show any cytotoxicity against the tumour cells. This state of anergy of intrinsic and regional lymphocytes is presumably acquired during the development of the cancer and would permit local tumour spread and metastasis to lymph nodes. Its cause has not been identified but appears to be lymphocyte inhibition rather than selective change in lymphocyte population. In particular, no special pattern can be seen in the relative proportions of T and B cells in patients' blood, lymph node or intrinsic carcinoma lymphocytes.


*Br. J. Cancer* (1973) **28**, 108

### LYMPHOCYTE ANERGY IN PATIENTS WITH CARCINOMA

A. P. P. NIND, R. C. NAIRN, J. M. ROLLAND, E. P. G. GULI AND E. S. R. HUGHES

*From the Department of Pathology, Monash University Medical School and Royal Melbourne Hospital, Melbourne, Australia*

Received 4 April 1973. Accepted 14 May 1973

**Summary.—All of 10 patients with colonic carcinoma and 5 with malignant melanoma of skin showed no sign of immunoreactivity against the cultured tumour cells by the lymphocyte populations residing within the tumours. More than half of these patients did show cytotoxic reactivity by their blood lymphocytes. Possible cytotoxic reactivity by the regional lymph node lymphocytes was also investigated in 57 tumour cases (44 colonic, 13 melanoma, and including 12 of the 15 examined for intrinsic lymphocyte activity). One third of the cases showed positive blood lymphocyte immunoreactivity, but in only 4 tumours (3 colonic) did the node lymphocytes show any cytotoxicity against the tumour cells. This state of anergy of intrinsic and regional lymphocytes is presumably acquired during the development of the cancer and would permit local tumour spread and metastasis to lymph nodes. Its cause has not been identified but appears to be lymphocyte inhibition rather than selective change in lymphocyte population. In particular, no special pattern can be seen in the relative proportions of T and B cells in patients' blood, lymph node or intrinsic carcinoma lymphocytes.**

In earlier reports of immunological reactivity in patients with carcinoma of colon (Nairn *et al.*, 1971*a*), and squamous cell carcinoma and malignant melanoma of skin (Nairn *et al.*, 1971*b*, 1972; Nairn, 1972), we observed that lymphocytes from the carcinoma itself or from regional lymph nodes were not cytotoxic against cultured tumour cells even though the patients' blood lymphocytes often were cytotoxic. Continued work has elaborated these preliminary observations and we have studied the phenomenon more deeply by analysing and varying, by cell fractionation procedures, the intrinsic lymphocyte populations of the carcinomata before culture. T and B cell proportions in the different lymphoid preparations have been assessed by membrane immunofluorescence and show no apparent correlation with lymphocyte cytotoxicity.

### MATERIALS AND METHODS

Tumour and blood collection, preparation of cell suspensions, freeze storage, inhibition of microbial growth, and culture of carcinoma cells for detecting cytotoxic activity of the patient's lymphocytes were all performed by the same techniques as in the earlier studies cited above.

For the investigation of intrinsic lymphocyte reactivity, 10 cases of colonic carcinoma and 5 of malignant melanoma of skin (2 metastatic) were selected only insofar as they were the first tumours received providing sufficient carcinoma cells and lymphocytes for the investigations. For most tests, the cells from the carcinoma were used fresh. Frozen cells preserved with dimethylsulphoxide in liquid nitrogen were used only when we were assured that this would not interfere with assessment of comparative tumour growth in culture. For the testing of possible interaction between intrinsic lymphocytes and blood or lymph node lymphocytes, the majority of blood and lymph node preparations were also used fresh for culture; only 1 lymphocyte specimen from blood and 3 from lymph node, all colonic cases, were freeze stored before use. The lymph nodes and blood lymphocytes were obtained about the time of operation, except for Case 6 in which blood lymphocytes were obtained 6 and 10 weeks later.

For the investigation of regional lymph node lymphocyte reactivity, 57 tumours were examined: 44 were colonic carcinomata (1 metastatic in liver) and 13 skin melanomata (4 metastatic in lymph node, 2 in brain), including all the cases in Table I except 3, 14, 15. In 31 cases, the lymphocytes were used fresh without prior freezing and the majority of the parallel blood lymphocyte cytotoxicity studies were also made with fresh cells (frozen–thawed blood lymphocytes were used in 11 colonic cases only). In 5 cases, the fresh blood lymphocytes were not available until some weeks after the operation but none were used beyond 20 weeks. Persistence of the patient's lymphocyte reactivity for several months after tumour resection has been established in our earlier and present studies. We were unable to test blood lymphocyte reactivity in 6 of the colonic carcinomata.

Spleen cell reactivity was investigated in 3 cases. Two were post mortem melanomata (12b, 13) and one a metastatic colonic carcinoma in liver (72/158—Table III).

*Tumour cell fractionation to alter intrinsic lymphocytosis.*—For this investigation, the 15 tumour cell suspensions plus 3 additional preparations from further specimens of 2 of the melanoma cases (11 and 12—Table I) were passed successively through and eluted from 2 sterile glass bead columns (modified from Shortman, 1966) to change the proportion of lymphocytes to carcinoma cells in the effluents. Samples of each effluent were kept for culture and wherever possible for T and B lymphocyte analysis.

The columns were made from 5 ml plastic syringe barrels with the nozzle lightly plugged with glass wool and fitted with a No. 22 Luer hypodermic needle; they contained 2 ml of glass beads of 60–90 $\mu$m diameter.

The original tumour cell suspension was gently washed 3 times in medium 199 containing 10% foetal calf serum with centrifuging at 250 *g* maximum for 5 min periods. It was adjusted to a concentration of 3–10 million cells per ml, and 5 ml of this suspension was passed through a glass bead column previously rinsed with medium 199 containing 10% foetal calf serum: this yielded fraction 1. The beads were then removed from the column and rinsed twice with 10 ml of the same medium to give fraction 2. Of this, 15–25 million cells were recovered by centrifuging and resuspended in 5 ml medium 199 containing 50% foetal calf serum and passed through a column previously rinsed with the same solution. This yielded fraction 3, and two 10 ml washings of the beads with the same medium, fraction 4. The viability of lymphocytes obtained in each fraction was always above 50%, as judged by exclusion of 0·1% trypan blue dye.

*Lymphocyte preparations from blood, lymph node and spleen.*—Blood lymphocytes were separated routinely for cytotoxicity tests by allowing heparinized blood to settle, and washing the buffy layer of leucocytes through a glass wool column with medium 199 containing 10% foetal calf serum. When prepared in this simple way, there was a small contamination by up to 10% granulocytes but accurate immunoglobulin-negative and -positive lymphocyte (respectively regarded here T and B cell) counts could usually be made without difficulty. In order to confirm this, many comparative counts were also made on blood lymphocyte preparations of greater purity obtained by more stringent separation procedures with glass beads or glass wool and Hypaque–Ficoll sedimentation of whole blood (*e.g.*, Yamana, Rolland and Nairn, 1973).

Lymph node lymphocytes and spleen cells were obtained in suspension by gentle mechanical teasing in medium 199 containing 10% foetal calf serum.

*Assay of T and B lymphocytes.*—This was by membrane immunofluorescence (Papamichail, Brown and Holborow, 1971). Fresh or frozen–thawed lymphocyte suspensions were treated with fluorescein-conjugated anti-human-globulin. This had activity against IgG, IgM and IgA, and a fluorescein to protein molar ratio of 4·9 : 1. It was absorbed with bovine liver and human group AB erythrocytes, and when used at a globulin concentration of 0·4% gave no fluorescent staining of T cells, as represented by infant thymocytes in preliminary studies. With this reagent, B cells gave bright membrane immunofluorescence; some lymphoid cells from tumour or lymph node gave solid cytoplasmic staining and were counted as B cells producing antibody.

Lymphocytes, numbering 100–200, were counted in each stained preparation, the B

TABLE 1.—*Effect of Varying Intrinsic Lymphocyte Content on Tumour Growth*

| | | | Tumour cell fractions | | | | | | | | | |
|---|---|---|---|---|---|---|---|---|---|---|---|---|
| | | | Original suspension | | Fraction 1 | | Fraction 2 | | Fraction 3 | | Fraction 4 | |
| Case and Record No. | Blood lymphocytes | | Tumour growth | Lymphocytes % | Tumour growth | Lymphocytes % | Tumour growth | Lymphocytes % | Tumour growth | Lymphocytes % | Tumour growth | Lymphocytes % |
| *Ca Colon* | | | | | | | | | | | | |
| 1 72/123 | Cytotoxic | (7) | 40 | 5 (40*) | 40 | 7 (44*) | 40 | 2 (53*) | 40 | 8 | 30 | 1 |
| 2 72/132 | Cytotoxic | (12) | 50 | 20 (29) | 80 | 40 (13) | 80 | 10 (20) | — | — | — | — |
| 3 72/122 | Cytotoxic | (15) | 30 | 2 (35*) | 40 | 5 | 30 | 1 | — | — | — | — |
| 4 72/88 | Cytotoxic | — | 50 | 10 (23*) | 60 | 25 | 60 | < 5 | 60 | 10 | 30 | < 5 |
| 5 72/7 | Cytotoxic | — | 20 | 10 (43*) | 30 | 20 | 30 | < 3 | 20 | 3 | 50 | 1 |
| 6 72/62 | Cytotoxic later | — | 30 | 10 (13*) | 30 | 30 | 30 | < 5 | 30 | 8 | 30 | < 1 |
| 7 72/115 | Non-cytotoxic | (2) | 40 | 4 (22*) | 30 | 10 (19*) | 50 | 1 | 50 | 3 | 50 | < 1 |
| 8 72/149 | Non-cytotoxic | (24) | 40 | 3 (15*) | 40 | 8 (19*) | 40 | 2 (14*) | 40 | 4 | 40 | 2 |
| 9 72/11 | Non-cytotoxic | — | 50 | 15 (16*) | 70 | 20 | 50 | 5 | — | — | — | — |
| 10 72/100 | Non-cytotoxic | (26) | 30 | 3 (31*) | 50 | 15 | 40 | 3 | 30 | 5 | 30 | 1 |
| *Melanoma* | | | | | | | | | | | | |
| 11a 72/63 | Cytotoxic | (18) | 30 | 30 (16) | 30 | 60 | 70 | 5 | 30 | 90 | 40 | < 5 |
| 11b | Cytotoxic | (18) | 60 | 10 (16) | 30 | 30 | 60 | < 5 | — | — | — | — |
| 11c | Cytotoxic | (18) | 50 | 30 | 30 | 50 | 50 | < 5 | — | — | — | — |
| 12a 72/37 | Cytotoxic | (24) | 40 | 30 (11) | 40 | 30 | 40 | 10 | 50 | < 5 | 30 | < 5 |
| 12b† 72/153 | Cytotoxic | (31) | 40 | 10 | 40 | 16 | 30 | 5 | 30 | 7 | 30 | 3 |
| 13† 72/128 | Cytotoxic | (26) | 30 | 3 (47*) | 80 | 5 (48*) | 50 | 1 | 30 | 4 | 90 | 2 |
| 14 70/87 | — | — | 50 | 30 (4) | 50 | 50 | 40 | 5 | — | — | — | — |
| 15 71/38 | — | — | 30 | 15 | 80 | 80 | — | 5 | 50 | 90 | 30 | < 5 |

" Tumour growth " figures indicate percentage area of culture chamber coverglass occupied by cancer cells.

Figures in brackets = % B cells.

* Includes many cells with cytoplasmic antibody.

† Post mortem cases.

cells were enumerated and the T cells assessed by difference. Accurate counting necessitated the recognition and ignoring of non-lymphoid cells usually unstained but occasionally stained nonspecifically, as in the case of granulocytes. These were largely removed from blood and fractionated carcinoma by their adherence to the glass wool and beads to which they were respectively exposed; unfractionated carcinoma cell suspensions contained only a few granulocytes and the lymph node preparations virtually none. The validity of our blood lymphocyte separation and discrimination by simple glass wool filtration was established by the comparisons with the alternative procedures already referred to. The cell populations of all specimens were confirmed morphologically by phase-contrast microscopy and identification of lymphocytes corresponded with immunofluorescent staining by specific antilymphocyte globulin.

*Lymphocyte reactivity against tumour in culture.*—Tumour cell suspensions were cultured, with or without test lymphocytes, in Teflon ring chambers mounted on standard microscope slides as in our earlier investigations already cited. The original tumour cell suspension ($3 \times 10^5$ cells in 0·6 ml culture medium 199 and 10% foetal calf serum plus 0·1 ml human blood group AB serum rich in complement) with its intrinsic lymphocyte population was cultured alone and with added test lymphocytes ($6 \times 10^5$) from patient's blood, lymph node or spleen, or as controls with homologous lymphocytes. Because the tumour cell suspensions contained a small proportion of stromal cells, the final ratio of added lymphocytes per tumour cell was approximately 2·5 : 1. The 4 tumour cell suspension fractions were similarly cultured in some experiments with added blood or lymph node lymphocytes from the patient. In a few experiments, cytotoxic lymphocytes were removed from larger culture chambers, purified by filtration through glass wool and retested in standard chambers in the same proportions against new tumour cells.

For cytotoxicity testing, the culture chamber was first incubated with coverglass down for 2 days and then inverted to facilitate growth of a monolayer on the coverglass, now uppermost, free from dead cells and debris which sink to the bottom of the chamber. Growth of tumour cells on the coverglasses of triplicate culture chambers was assessed microscopically after 5 days (Nairn *et al.*, 1971*a*) as the percentage area occupied by viable tumour cells, to the nearest 10%. In the absence of cytotoxic effect, the monolayer covered 50–100% of the coverglass within this period. Mean values of the replicates, for test and control cultures, read blind, were compared in every experiment and a difference of over 20% was required for significant cytotoxicity assessment (Table II). With this test system, estimating lymphocytotoxicity is mostly an "all or none" observation, since in the vast majority of experiments where cytotoxicity was demonstrated the test cultures showed virtually no viable cancer cells on the coverglasses as opposed to a 50–100% area coverage in the normal control cultures. In Table I, mean values for triplicate cultures of the original tumour suspension and its fractions are given. Variation between replicates in all experiments were small, hardly ever greater than 10% area.

### RESULTS

### *Intrinsic lymphocytes*

Varying the intrinsic lymphocyte population of the different tumour fractions had no significant effect on tumour cell proliferation (Table I). The variations in growth that occurred were not consistently correlated with the lymphocyte numbers or T and B cell proportions. The proportion of lymphocytes in the cultures in all but 2 cases (11a and 15) was lower than the 70% routinely employed for blood lymphocyte cytotoxicity testing although it would be reasonable to expect that such intrinsic lymphocytes might include a larger proportion specifically immune to the tumour.

Blood lymphocyte reactivity with tumour fractions (Table II) was tested in 4 colonic carcinomata (Cases 4, 6, 7, 8) and 2 specimens of 1 melanoma (Case 12a, b). In Cases 4, 7 and 8, tests were made only against the original tumour and fraction 1, whereas in the others all fractions were tested. The variation in lymphocyte population had no effect on the reactivity of either positive or negative

TABLE II.—*Effect of Patients' Lymphocytes on Tumour Growth*

| Case or Record No. | Tumour alone | With patients' lymphocytes: Blood† | Regional lymph node | Distant lymph node | Spleen | With homologous lymphocytes |
|---|---|---|---|---|---|---|
| *Ca Colon* | | | | | | |
| 72/111 | 50 | < 10 | 30 | — | — | 50 |
| 6* | 30 | 40* | 10 | 40 | — | 40 |
| 6 (Fraction 1) | 30 | 50 | — | — | — | 50 |
| 6 (Fraction 2) | 30 | 40 | — | — | — | 30 |
| 6 (Fraction 3) | 30 | 50 | — | — | — | 50 |
| 6 (Fraction 4) | 30 | 50 | — | — | — | 50 |
| 72/158 | 60 | 60 | 40 | — | 20 | 60 |
| 1 | 40 | 20 | 50 | — | — | 40 |
| 2 | 50 | 30 | 60 | — | — | 60 |
| 4 | 50 | < 10 | 50 | — | — | 50 |
| 4 (Fraction 1) | 60 | < 10 | 70 | — | — | 70 |
| 4 (Fraction 2) | 60 | — | 70 | — | — | — |
| 4 (Fraction 3) | 60 | — | 50 | — | — | — |
| 4 (Fraction 4) | 30 | — | 30 | — | — | — |
| 5 | 20 | 0 | 20 | — | — | 20 |
| 70/80 | 90 | 10 | 90 | — | — | 100 |
| 72/81 | 50 | < 10 ( < 10) | 40 | — | — | 50 |
| 72/92 | 40 | < 10 | 40 | 40 | — | 40 |
| 72/109 | 30 | 10 | 40 | — | — | 40 |
| 72/125 | 30 | < 10 | 30 | — | — | 30 |
| 3 | 30 | < 10 | — | — | — | 30 |
| 7 | 40 | 40 | 30 | — | — | 40 |
| 7 (Fraction 1) | 30 | 30 | 30 | — | — | 30 |
| 8 | 40 | 50 | 50 | — | — | 50 |
| 8 (Fraction 1) | 40 | 60 | — | — | — | 40 |
| 9 | 50 | 60 | 60 | — | — | 60 |
| 10 | 30 | 40 | 30 | — | — | 30 |
| 71/137 | 50 | 50 | 50 | — | — | 50 |
| 72/9 | 70 | 70 (70) | 70 | — | — | 70 |
| 72/13 | 30 | 20 | 30 | — | — | — |
| 72/14 | 50 | 50 | 50 | — | — | — |
| 72/15 | 20 | 30 | 30 | — | — | 20 |
| 72/16 | 20 | 20 | 20 | 30 | — | 20 |
| 72/20 | 30 | 40 | 30 | — | — | — |
| 72/22 | 60 | 60 | 50 | — | — | 60 |
| 72/23 | 20 | 20 | 20 | — | — | 20 |
| 72/25 | 70 | 60 | 60 | — | — | 60 |
| 72/26 | 50 | 50 | 50 | — | — | — |
| 72/29 | 50 | 60 (60) | 50 | — | — | 50 |
| 72/48 | 40 | 50 (40) | 50 | — | — | 40 |
| 72/52 | 20 | 30 | 30 | 30 | — | 30 |
| 72/61 | 40 | 50 | 40 | — | — | 40 |
| 72/94 | 30 | 30 | 30 | — | — | 30 |
| 72/102 | 20 | 40 | 20 | — | — | 20 |
| 72/118 | 30 | 40 | 30 | — | — | 30 |
| 72/119 | 20 | 20 | 20 | — | — | 20 |
| 72/137 | 30 | 30 | 30 | 30 | — | 30 |
| 72/139 | 40 | 30 | 30 | 30 | — | 40 |
| 72/154 | 60 | 60 | 60 | — | — | 50 |
| 71/20 | 80 | — | 70 | 80 | — | 80 |
| 71/139 | 70 | — | 70 | — | — | — |
| 72/38 | 30 | — | 20 | 20 | — | 20 |
| 72/82 | 50 | — | 50 | — | — | 50 |
| 72/121 | 20 | — | 20 | — | — | 20 |
| 72/150 | 60 | — | 70 | 70 | — | 70 |
| *Malignant Melanoma* | | | | | | |
| 11a | 30 | < 10 | 10 | — | — | 50 |
| 11a (Fraction 1) | 30 | — | 10 | — | — | 60 |
| 11b | 60 | 20 | 40 | — | — | — |
| 11b (Fraction 2) | 60 | — | 30 | — | — | — |
| 12a | 40 | < 10 | — | — | — | 70 |

TABLE II.—(*continued*)

| Case or Record No. | Tumour alone | With patients' lymphocytes: Blood† | Regional lymph node | Distant lymph node | Spleen | With homologous lymphocytes |
|---|---|---|---|---|---|---|
| 12a (Fraction 1) | 40 | < 10 | — | — | — | 70 |
| 12a (Fraction 2) | 40 | 10 | — | — | — | 70 |
| 12a (Fraction 3) | 50 | < 10 | — | — | — | 70 |
| 12a (Fraction 4) | 30 | < 10 | — | — | — | — |
| 12b | 40 | 10 | 50 | 40 | 50 | 50 |
| 12b (Fraction 1) | 40 | 20 | — | — | — | 50 |
| 12b (Fraction 2) | 30 | 10 | — | — | — | 50 |
| 12b (Fraction 3) | 30 | 10 | — | — | — | 50 |
| 12b (Fraction 4) | 30 | 10 | — | — | — | 50 |
| 13 | 30 | < 10 | 40 | 40 | 40 | 40 |
| 71/59 | 90 | 50 | 90 | — | — | 100 |
| 72/96 | 50 | < 10 ( < 10) | 50 | — | — | 50 |
| 71/133 | 90 | 50 | 90 | — | — | 90 |
| 71/31 | 90 | 90 | 90 | — | — | 90 |
| 71/67 | 70 | 70 | 70 | — | — | 80 |
| 71/117 | 80 | 70 | 70 | — | — | 80 |
| 71/125 | 70 | 80 | 70 | — | — | 70 |
| 70/96 | 50 | 50 | 50 | — | — | 50 |
| 72/93 | 20 | 30 | 30 | — | — | 30 |
| 72/34 | 30 | 30 (30) | 30 | — | — | 30 |

Figures indicate percentage area of culture chamber coverglass occupied by cancer cells.
† Figures in brackets indicate tumour growth obtained after addition of blood and lymph node lymphocytes together.
* Case 6 blood lymphocytes became positive ( < 10) 10 weeks post-operation.

blood lymphocytes. Six tests were made with non-cross-reacting homologous lymphocytes against colonic carcinoma (4) and melanoma (2) fractions without any change in reactivity.

Lymph node lymphocyte reactivity with tumour fractions (Table II) was tested in 2 colonic carcinomata (Cases 4, 7) and 2 specimens of 1 melanoma (11a,b). In Case 4, all fractions were tested, in 7 and 11a only fraction 1, and in 11b fraction 2. Reactivity against the colonic carcinomata was negative and unchanged with the fractions; against the melanoma specimens it was positive and also unchanged with the fractions.

*Lymph node lymphocytes*

Only 4 of the 57 tumours examined showed reactivity by the regional lymph node lymphocytes (Table II): 3 were colonic including 1 liver metastasis (Cases 6, 72/111, 72/158) and 1 a melanoma (Case 11). The blood lymphocytes at the same lymphocyte to tumour cell ratio in the cultures (2·5 : 1) showed positive cytotoxicity in 17 of the 51 cases in which satisfactory tests were possible. Of the 4 positive node lymphocyte cytotoxicity experiments, Case 6 was at first blood lymphocyte-negative but became positive with a repeat blood specimen 4 weeks later, Case 11 was positive in repeated tests; Case 72/111 was also positive and Case 72/158 negative. The positive blood lymphocyte reactivity was always stronger than that of the node lymphocytes.

Six experiments were performed to investigate possible interaction between lymph node and blood lymphocytes (Table II): 4 were with colonic carcinomata and 2 melanomata, none being from the cases recorded in Table I. One of the colonic and 1 melanoma case were blood lymphocyte-positive; they were all lymph node lymphocyte-negative. For these tests, the blood and lymph node lymphocytes were added to the tumour culture chambers in equal numbers to give the usual lymphocyte to tumour cell ratio of 2·5 : 1. There was no sign of any effect by lymph node lymphocytes on the already known blood lymphocyte reactivity.

In 11 of the cases (9 colonic), lymph nodes distant from the tumour were also examined (Table II). The lymphocytes were tested fresh except in 4 of the colonic carcinomata where they were frozen–thawed. Both of the melanoma cases and 1 colonic were blood lymphocyte-positive; another colonic carcinoma (Case 6) was blood lymphocyte-positive later and had positive regional (near) node lymphocyte reactivity. Blood and regional node lymphocyte reactivities were negative in the other cases. In all 11 cases, the distant node lymphocytes were non-reactive.

T and B cell counts were made on 25 of the regional lymph nodes, on 16 of the associated tumours and on 12 of the relevant blood samples and 7 of the distant nodes. The findings in the 16 cases in which both regional lymph node and tumour were examined are summarized in Table III. It will be seen that except in

TABLE III.—*B Lymphocyte Proportions in Blood, Regional Lymph Node and Tumour*

| Case or Record no. | Blood lymphocytotoxicity | B cells % Blood | Lymph node | Tumour |
|---|---|---|---|---|
| *Ca Colon* | | | | |
| 72/111 | Cytotoxic | 26 | 36† | 21* |
| 6 | Cytotoxic (later) | 1 (later) | 19† | 13* |
| 1 | Cytotoxic | 7 | 39 | 51* |
| 2 | Cytotoxic | 12 | 25 | 29 |
| 72/125 | Cytotoxic | 22 | 42 | 43* |
| 72/158 | Non-cytotoxic | 2 | 13† | 64* |
| 7 | Non-cytotoxic | 2 | 32 | 22* |
| 72/139 | Non-cytotoxic | 26 | 30 | 33* |
| 72/118 | Non-cytotoxic | — | 36 | 32* |
| 72/48 | Non-cytotoxic | — | 85* | 8* |
| 10 | Non-cytotoxic | 26 | 32 | 31* |
| *Melanoma* | | | | |
| 11 | Cytotoxic | 18 | 38† | 16 (a,b) |
| 13 | Cytotoxic | 26 | 52 | 47 |
| 12b | Cytotoxic | 31 | 9 | ? |
| 72/96 | Cytotoxic | — | 52 | 28 |
| 72/93 | Non-cytotoxic | — | 20 | 14 |

* Includes many cells with cytoplasmic antibody.
† Lymphocytotoxic-positive.
? Uncountable because of confusion by pre-existing globulin-binding to tumour cells.

1 case of melanoma (12b), the B lymphocyte count in the blood was lower than in the regional node and, except for 2 lymph node-positive cases (72/111, 11), than in the tumour itself; the node count was commonly a little higher than the tumour. Solid cytoplasmic immunofluorescent staining cells, considered to be antibody forming, were found only among the intrinsic lymphocytes of the colonic carcinomata except for 1 regional node (Case 72/48) which showed a very high B cell count (85% of total lymphocytes); the blood lymphocyte count is not available from this case. The abundance of antibody forming cells in the colonic tumours is probably attributable to their greater exposure to infection. The blood B lymphocyte counts were frequently outside our normal range (8–24%) obtained with the present technique on 10 normal individuals. This wider variation of T and B cell counts is presumably largely attributable to patients' immunological reactivities to the tumour itself, its necrotic products, or its microbial contaminants.

### *Spleen cells*

Tumour cytotoxicity studies were made on spleen cell suspensions (Table II) in the 2 post mortem melanoma cases (12b, 13) both examined within 10 hours of death: results were negative despite the post mortem blood lymphocytes being cytotoxic to tumour cells. Their failure to react may not necessarily reflect their cytotoxic capability during life, because of possible selective death of immune cells immediately post mortem. A third case (72/158) of metastatic liver tumour from a primary colonic carcinoma, tested against spleen cells obtained during life, showed strong cytotoxicity, hepatic lymph node lymphocytes less cytotoxicity, and the blood lymphocytes were non-reactive.

B cell counts on the 3 spleens were unremarkable, being respectively 22, 37 and 23%.

### *Cytotoxic lymphocytes re-reacted on new tumour cells*

Preliminary studies to assess whether cytotoxic blood lymphocytes or spleen cells retain any reactivity after 5 days' cultivation with tumour were made in 2 melanoma cases (12a, 72/136) and 1 colonic case (72/158). The larger original culture chambers with the usual cell proportions yielded sufficient supernatant for a second tumour cytotoxicity test by the routine procedure, but there was no remaining cytotoxicity by the re-used lymphocytes.

*Immunopathological correlation*

No correlation has been observed between lymphocyte reactivity against the tumours and their histopathological differentiation or dissemination or the immediate prognosis of the patients. The short-term fate has been followed in 42 patients including 37 colonic carcinomata and 5 melanomata. Death has occurred within 6 months from operation in all of 3 blood lymphocyte-positive resected melanoma patients, including 1 lymph node-positive. Of the colonic carcinoma patients, 1 of 10 blood lymphocyte-positive, 4 of 27 blood lymphocyte-negative, including 1 lymph node-positive, were dead within 8 months from operation.

On the other hand, blood and lymph node lymphocyte reactivities have been associated to some extent with particular lymphoid histological patterns in tumour and lymph node respectively. Twenty of 25 blood lymphocyte-negative colonic cases showed a diffuse stromal leucocytic infiltration of the tumours including large lymphocytes and plasma cells associated with eosinophil granulocytes. This type of infiltration was found in only 4 of 8 lymphocyte-positive cases, which instead showed a tendency to discrete perivascular aggregation of small hyperchromatic lymphocytes. Such aggregates were the sole type of reaction in 2 of the positive cases and were never conspicuous in the negative cases. The 3 positive lymph nodes from colonic carcinomata all showed, histologically, a diffuse increase of small lymphocytes with follicular hypoplasia and inconspicuous or absent germinal centres. This pattern was not observed in any of 36 lymphocyte-negative lymph nodes examined: in these there was only nondescript follicular and/or sinus hyperplasia. No notable histological features were recognized in the 3 spleens.

### DISCUSSION

We have observed no material *in vitro* cytotoxic activity against colonic carcinomata or melanomata by their intrinsic lymphocyte populations. Moreover, in only 4 regional nodes of 57 cases examined was weak lymphocyte reactivity demonstrated. In contrast, there was an overall positive blood lymphocyte reactivity in a third of cases. Local lymph node lymphocyte anergy has also been reported in human tumours by DiSaia *et al.* (1971) and Vánky and Stjernswärd (1971), but we have seen no systematic study of the phenomenon. The timing of the anergy is in doubt, but experimental studies in this laboratory on a rat squamous cell carcinoma (Flannery *et al.*, 1973) showed that regional nodes are immunoreactive for some weeks and then become nonreactive. Landazuri and Herberman (1972) also report a waxing and waning of lymphocyte cytotoxicity in a rat virus lymphoma system.

The maintainance of blood lymphocyte cytotoxicity and the source of the effector cells are further problems for study. Among the possible tissue sites of antitumour lymphocyte proliferation are the tumour stroma, lymph nodes, spleen or bone marrow. Only the last has not been examined at all in this study, and although current immunological theory makes it an unlikely source of cytotoxic lymphocytes, it deserves investigation. More likely sources of the cytotoxic blood lymphocytes are the local, intrinsic, regional lymph node and perhaps spleen populations, despite their frequent anergy at the time of testing. In support are our observations that positive blood lymphocyte cytotoxicity is correlated histopathologically with tumour infiltration by discrete aggregates of small lymphocytes, that some regional nodes contain reactive lymphocytes and that 1 case had reactive spleen cells.

Tumour infiltration by lymphocytes has been reported as a favourable prognostic feature in breast (Hamlin, 1968) and melanoma (Thompson, 1972) though other studies have failed to observe any such correlation (Williams and Roberts, 1968; Little, 1972). Whatever effect there might be on prognosis, it cannot be great, and would perhaps reflect an active state of the infiltrating lymphocytes at an earlier stage before the anergy reported here had developed.

It may be speculated that resident lymphocytes within the tumour and the local lymph nodes become inactivated in some way to be determined, while those that escape the local environment and reach the circulation retain or regain their specific tumour cytotoxicity. Could this be lymphocyte washing by circulation in non-inhibiting serum (*cf.* Currie and Basham, 1972)? However, 1 of our colonic cases (72/158) was blood lymphocyte-negative at a time when the lymph node was positive. We found no sign of collaboration of local lymphocytes with blood lymphocytes, which would seem to make untenable one explanation of regaining of cytotoxicity by any such lymphocyte collaboration. Another theoretical explanation of our findings, namely a failure of immunoglobulin-positive and -negative ("B and T") lymphocyte collaboration by variation in their proportions at local sites, is supported by the observation that T cells are usually more numerrous in the blood, but we have examples of high T cell (low B cell) counts in the instrinsic tumour or regional lymph node lymphocytes without evidence of tumour cytotoxicity.

The possibility must be considered that local killer lymphocytes may themselves be destroyed or lose their killer potentiality on contact with the tumour or its antigens, leaving immunoreactive only those which escape to the blood. Our failure to detect any recycling of lymphocyte cytotoxicity in the 3 repeat cultures studied of used lymphocytes against fresh tumour cells favours this. However, these experiments are too few for ready acceptance; indeed the occurrence of such lymphocyte recycling has been reported in an experimental system by Berke, Sullivan and Amos (1972).

Specific blocking of *in vitro* lymphocyte cytotoxic attack on human cells by the serum of cancer patients (Hellström *et al.*, 1971) may be mediated by antigen–antibody complexes (Sjögren *et al.*, 1972), probably reacting primarily with sensitized lymphocytes (Currie and Basham, 1972). Blocking reactivity of sera from our cases has not yet been investigated, but detection of antibodies against tumour cell membrane and cytoplasm (Rolland *et al.*, to be published) correlated poorly with lymphocyte cytotoxicity. Studies on an experimental animal tumour system in our laboratories have demonstrated inhibitory activity in sera from animals bearing advanced tumours at a time when regional lymph nodes were anergic despite persistent blood and spleen lymphocyte cytotoxicity (Flannery *et al.*, 1973). This is presumably due to prolonged exposure of local lymphocytes to relatively high concentrations of soluble antigen, causing a specific immunological paralysis (Alexander, 1970; Baldwin, Price and Robins, 1972).

Whatever the cause of the local lymphocyte anergy, the phenomenon is presumably highly significant biologically and may be the explanation of tumour spread and regional metastasis despite local abundance of lymphocytes. It will be of great interest to examine, wherever clinically permissible, lymph nodes from the tumour drainage area some months after tumour resection to see if the anergic state may be reversed.

This work was supported by grants from the Anti-Cancer Council of Victoria and the National Health and Medical Research Council. For sending us specimens we thank Dr N. C. Davis and Dr J. H. Little of the Queensland Melanoma

Project; Dr A. V. Jackson and Mr A. J. Rollo of Alfred Hospital; Dr J. F. Funder of Prince Henry's Hospital; Professor M. R. Ewing, Mr A. M. Cuthbertson, Mr I. S. Russell and Mr M. Long, of the Royal Melbourne Hospital; and Mr G. W. Trinca of Preston and Northcote Hospital. We also thank Miss P. D. Chatfield, Miss H. C. Goldsmith, Miss E. L. Jakimoff, Mr D. Quinn and Mr J. N. Soar for technical assistance.

### REFERENCES

ALEXANDER, P. (1970) Prospects for Immunotherapy of Cancer: Experience in Experimental Systems. *Br. med. J.*, iv, 484.

BALDWIN, R. W., PRICE, M. R. & ROBINS, R. A. (1972) Blocking of Lymphocyte-mediated Cytotoxicity for Rat Hepatoma Cells by Tumour-specific Antigen–Antibody complexes. *Nature, New Biol.*, **238**, 185.

BERKE, G., SULLIVAN, K. A. & AMOS, D. B. (1972) Tumour Immunity *in vitro*: Destruction of a Mouse Ascites Tumour Through a Cycling Pathway. *Science, N.Y.*, **177**, 433.

CURRIE, G. A. & BASHAM, C. (1972) Serum Mediated Inhibition of the Immunological Reactions of the Patient to His Own Tumour: A Possible Role for Circulating Antigen. *Br. J. Cancer*, **26**, 427.

DISAIA, P. J., RUTLEDGE, F. N., SMITH, J. P. & SINKOVICS, J. G. (1971) Cell-mediated Immune Reaction to Two Gynecologic Malignant Tumors. *Cancer, N.Y.*, **28**, 1129.

FLANNERY, G. R., CHALMERS, P. J., ROLLAND, J. M. & NAIRN, R. C. (1973) Immune Response to a Syngeneic Rat Tumour: Development of Regional Node Lymphocyte Anergy. *Br. J. Cancer*, **28**. In the press.

HAMLIN, I. M. E. (1968) Possible Host Resistance in Carcinoma of the Breast: a Histological Study. *Br. J. Cancer*, **22**, 383.

HELLSTRÖM, I., HELLSTRÖM, K. E., SJÖGREN, H. O. & WARNER, G. A. (1971) Serum Factors in Tumor-free Patients Cancelling the Blocking of Cell-mediated Tumor Immunity. *Int. J. Cancer*, **8**, 185.

DE LANDAZURI, M. O. & HERBERMAN, R. B. (1972) Immune Response to Gross Virus-induced Lymphoma. III. Characteristics of the Cellular Immune Response. *J. natn. Cancer Inst.*, **49**, 147.

LITTLE, J. H. (1972) Histology and Prognosis in Cutaneous Malignant Melanoma. In *Melanoma and Skin Cancer*. I.U.A.C. Proc. Int. Cancer Conf., Sydney. Government printer. p. 107.

NAIRN, R. C. (1972) Mechanisms, Detection and Significance of Immunity to Skin Cancer. In *Melanoma and Skin Cancer*. I.U.A.C. Proc. Int. Cancer Conf., Sydney. Government printer. p. 257.

NAIRN, R. C., NIND, A. P. P., GULI, E. P. G., DAVIES, D. J., ROLLAND, J. M., MCGIVEN, A. R. & HUGHES, E. S. R. (1971*a*) Immunological Reactivity in Patients with Carcinoma of Colon. *Br. med. J.*, iv, 706.

NAIRN, R. C., NIND, A. P. P., GULI, E. P. G., MULLER, H. K., ROLLAND, J. M. & MINTY, C. C. J. (1971*b*) Specific Immune Response in Human Skin Carcinoma. *Br. med. J.*, iv, 701.

NAIRN, R. C., NIND, A. P. P., GULI, E. P. G., DAVIES, D. J., LITTLE, J. H., DAVIS, N. C. & WHITEHEAD, R. H. (1972) Anti-tumour Immunoreactivity in Patients with Malignant Melanoma. *Med. J. Aust.*, **1**, 397.

PAPAMICHAIL, M., BROWN, J. C. & HOLBOROW, E. J. (1971) Immunoglobulins on the Surface of Human Lymphocytes. *Lancet*, ii, 850.

SHORTMAN, K. (1966) The Separation of Different Cell Classes from Lymphoid Organs. I. The Use of Glass Bead Columns to Separate Small Lymphocytes, Remove Damaged Cells and Fractionate Cell Suspensions. *Aust. J. exp. Biol. med. Sci.*, **44**, 271.

SJÖGREN, H. O., HELLSTRÖM, I., BANSAL, S. C., WARNER, G. A. & HELLSTRÖM, K. E. (1972) Elution of "Blocking Factors" from Human Tumors, Capable of Abrogating Tumor-cell Destruction by Specifically Immune Lymphocytes. *Int. J. Cancer*, **9**, 274.

THOMPSON, P. G. (1972) The Relationship of Lymphocyte Infiltration to Prognosis in Primary Malignant Melanoma of Skin. 8th Internat. Pigment Cell Conf. Proc. Abstract, I.U.A.C. Sydney. p. 100.

VÁNKY, F. & STJERNSWÄRD, J. (1971) Tumor-distinctive Cellular Immunity to Human Sarcoma and Carcinoma. *Israel J. med. Sci.*, **7**, 211.

WILLIAMS, W. J. & ROBERTS, M. M. (1968) Delayed Hypersensitivity in Breast Cancer. In *Prognostic Factors in Breast Cancer*. Ed. A. P. M. Forrest and P. B. Kunkler. Edinburgh: Livingstone. p. 331.

YAMANA, S., ROLLAND, J. M. & NAIRN, R. C. (1973) T and B Cells in Various Lymphoid Tissues in Mice: Membrane Immunofluorescence with Purified Lymphoid Cells. *Immunological Communications*. In the press.